# Which Morphological Characteristics Are Most Influenced by the Host Matrix in Downy Mildews? A Case Study in *Pseudoperonospora cubensis*

**DOI:** 10.1371/journal.pone.0044863

**Published:** 2012-11-15

**Authors:** Fabian Runge, Beninweck Ndambi, Marco Thines

**Affiliations:** 1 University of Hohenheim, Institute of Botany, Stuttgart, Germany; 2 University of Hohenheim, Institute of Plant Production and Agroecology in the Tropics and Subtropics, Stuttgart, Germany; 3 Biodiversity and Climate Research Centre (BiK-F), Frankfurt (Main), Germany; 4 Senckenberg Gesellschaft für Naturforschung, Frankfurt (Main), Germany; 5 Johann Wolfgang Goethe University, Department of Biological Sciences, Institute of Ecology, Evolution and Diversity, Frankfurt (Main), Germany; Soonchunhyang University, Republic of Korea

## Abstract

Before the advent of molecular phylogenetics, species concepts in the downy mildews, an economically important group of obligate biotrophic oomycete pathogens, have mostly been based upon host range and morphology. While molecular phylogenetic studies have confirmed a narrow host range for many downy mildew species, others, like *Pseudoperonospora cubensis* affect even different genera. Although often morphological differences were found for new, phylogenetically distinct species, uncertainty prevails regarding their host ranges, especially regarding related plants that have been reported as downy mildew hosts, but were not included in the phylogenetic studies. In these cases, the basis for deciding if the divergence in some morphological characters can be deemed sufficient for designation as separate species is uncertain, as observed morphological divergence could be due to different host matrices colonised. The broad host range of *P. cubensis* (ca. 60 host species) renders this pathogen an ideal model organism for the investigation of morphological variations in relation to the host matrix and to evaluate which characteristics are best indicators for conspecificity or distinctiveness. On the basis of twelve morphological characterisitcs and a set of twelve cucurbits from five different Cucurbitaceae tribes, including the two species, *Cyclanthera pedata* and *Thladiantha dubia*, hitherto not reported as hosts of *P. cubensis*, a significant influence of the host matrix on pathogen morphology was found. Given the high intraspecific variation of some characteristics, also their plasticity has to be taken into account. The implications for morphological species determination and the confidence limits of morphological characteristics are discussed. For species delimitations in *Pseudoperonospora* it is shown that the ratio of the height of the first ramification to the sporangiophore length, ratio of the longer to the shorter ultimate branchlet, and especially the length and width of sporangia, as well as, with some reservations, their ratio, are the most suitable characteristics for species delimitation.

## Introduction

The family Peronosporaceae is the largest oomycete family and contains, in addition to smaller groups, the about 100 species of the paraphyletic, hemibiotrophic genus *Phytophthora* and the about 800 species of obligate biotrophic downy mildews [1]–[4]. Among them are several economically important diseases, like *Phytophthora infestans* (potato late blight), *Bremia lactucae* (lettuce downy mildew), *Plasmopara halstedii* (sunflower downy mildew), *Plasmopara viticola* (grape downy mildew), and *Pseudoperonospora cubensis* (cucurbit downy mildew). As it is often difficult to distinguish downy mildew species on the basis of morphological characters, Yerkes & Shaw [5] favoured a broad species concept for two large groups of downy mildews of Chenopodiaceae and Brassicaceae, respectively. This was contrasting the narrow species concept advocated by Gäumann [6], [7], stating that host ranges of downy mildews are often limited to a single host species. Although many plant pathologists have adopted the broad species concept, Gäumann's concept has mostly been used by taxonomists and has largely been confirmed by molecular studies (e.g. [8]–[14]). But among obligate biotrophic oomycetes not only *Albugo candida* of the white blister rusts (Albuginales) has been demonstrated to have a broad host range [15]–[19], but also some downy mildew species, for example in *Hyaloperonospora* [14], *Bremia* [13], and *Pseudoperonospora* [20], [21]. *Pseudoperonospora cubensis*, which is one of the most important pathogens of cucurbitaceous crops, is unusual among downy mildews, however, because its reported host range encompasses about 60 species of cucurbitaceous plants in several tribes. The integrity of *P. cubensis* was seldom questioned, although Sawada [22] segregated new species from this species, based on differences in sporangial dimensions associated with some host plants. Although these new species were not considered by subsequent authors [23]–[26], the potential risk of misinterpreted morphological divergence or similarity is obvious.

Several studies have addressed the morphological variability of downy mildews, mainly regarding ecological conditions. Iwata [23] and Cohen & Eyal [27] showed that the morphology of sporangiophores of *P. cubensis* varies with different temperatures and intensity of light. Dudka et al. [28] reported humidity as a key environmental factor that has a major impact on sporangial dimensions of *Peronospora alta*. Delanoe [29] pointed out, that even the kind of host tissue from which sporulation takes place may affect the morphology of sporangiophores and sporangia of *Plasmopara halstedii*, as for instance the sporangia produced from roots were more than two times larger than the sporangia produced from leaves. Few studies are available that address the variability of the morphology of different pathogen isolates from the same species on the same host. Kulkarni et al. [30] found that the sporangiophores and also the sporangia of different isolates of *Plasmopara halstedii* varied significantly in their morphology when grown on the same host under controlled conditions. And Salati et al. [31] showed that the size of the sporangiophores of different *Pseudoperonospora cubensis* isolates taken from the same host can vary significantly. Morphology of downy mildews thus seems to be dependent on several different factors, some of which have not been characterized to date. Most of the comparative studies of closely related downy mildews underpin their molecular phylogenetic results with morphological differences of the pathogens, even if there are only very few diagnostic SNPs. Since these pathogens originate from different hosts and data from infection trials for both pathogens on the same host is mostly not available, it often cannot be assessed, whether downy mildews on closely related hosts could be potentially conspecific. However, Runge & Thines [32] demonstrated for the closely related species *P. cubensis* and *P. humuli* that two distinct species may look more dissimilar on different hosts compared to the differences between the two pathogens if the same host was infected. Thus morphologically cryptic, but phylogenetically distinct species might exist on the same host. Conversely, two isolates belonging to the same species could potentially be very dissimilar on two distinct hosts in terms of morphology. The impact of host matrix on sporangial dimensions and a limited number of additional characteristics has already been shown for *P. cubensis* [26], [33]. But it is currently unclear, how the broad set of characteristics currently used for species delimitation [8], [9], [12], [34] is influenced by the host matrix, and if increasing phylogenetic distance of the hosts would lead to stronger differences in the morphology of the pathogen, or if other factors are more important. In addition, it is important to assess which characters are most and least influenced by the host matrix to enable the identification of characters by which known species on new hosts could be reliably identified. Some hints regarding the variability of morphological characteristics on different hosts were obtained in our previous work [33], but the limited sampling of hosts among Cucurbitaceae and the few characters investigated were not sufficient to resolve these questions.

Based on twelve hosts and twelve morphological characteristics, the aim of this study was to evaluate, how plastic morphological characteristics used for species delimitation are, on a single host and comparing different host matrices.

## Materials and Methods

**Figure 1:**
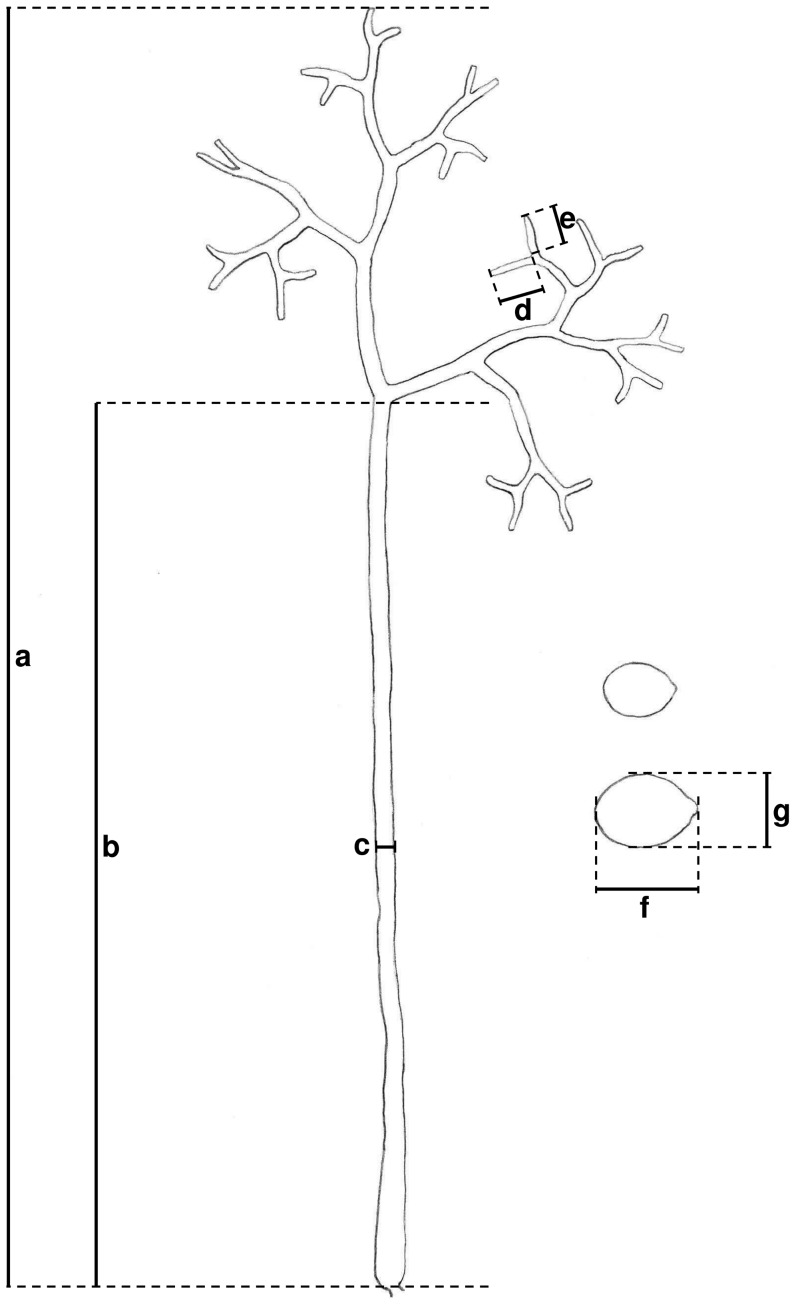
Drawing of a sporangiophore and sporangia of *Pseudoperonospora cubensis* isolated from *Cucumis sativus*. Measurements were taken for the length of the sporangiophore (a), height of the first ramification (b), width of the trunk (c), length of the longer (d) and shorter (e) ultimate branchlets, as well as the length (f) and the width (g) of sporangia.

Infection experiments were carried out using the *Pseudoperonospora cubensis* strain P.C. 26/01 that originally was isolated from *Cucumis sativus* in the Czech Republic. The strain is maintained as a reference strain on *C. sativus* in climate chambers (16°C, 14 h light, and 10 h darkness) since 2007 at the Institute of Botany at the University of Hohenheim. Inoculations for continuous cultivation of the strain and crossinoculations were done using the dab-off technique as described earlier [35]. Uninfected leaves were moistened on the lower leaf surface with deionised water, followed by gently dabbing sporulating leaf parts onto the moist leaf surface. The dab-off technique leads to a range of the spore concentration over the leaf surface from areas without sporangia to areas with very high amounts of sporangia. The advantage is that the optimal inoculum concentrations, which will differ from species to species and even from cultivar to cultivar, need not to be determined, because these will be met through the mosaic pattern in inoculum load throughout the surface of the leaves. Fully mature leaves of *Bryonia dioica*, *Citrullus lanatus*, *Cucumis anguria*, *C. melo*, *C. sativus*, *Cucurbita maxima*, *Cu. moschata*, *Lagenaria siceraria*, and *Luffa cylindrica* were taken from plants in 5- to 10-leaf stage grown in greenhouses at the University of Hohenheim. Additionally leaves of a comparable stage of *Cyclanthera pedata*, *Sicyos angulatus*, and *Thladiantha dubia* were taken from outdoor plants at the Botanical Garden of the University of Hohenheim. After inoculation the leaves were transferred to transparent boxes (approximately 30 cm long, 20 cm wide, 5 cm high) on water-soaked paper towels to ensure that 100% relative humidity (RH) was maintained within the boxes. Crossinoculations were done in three technical replicates. Crossinoculations using *B. dioica*, *Ci. lanatus*, *C. anguria*, *Cu. maxima*, *S. angulatus*, and *T. dubia* were in addition repeated at different time points for testing reproducibility over time. As these tests were successful and did not reveal significant differences, infection trials for the other cucurbitaceous hosts were done only once, in three replicates carried out at the same time. Two days after sporulation was first observed, sporangiophores were picked from the leaf surface with precision tweezers, transferred to a drop of water on a microscopic slide and covered with a coverslip. The morphology of sporangia and sporangiophores were investigated using a Biomed (Leitz, Wetzlar, Germany) light microscope. Pictures of sporangia and sporangiophores were taken using a Canon PowerShot A640 camera (Canon, Tōkyō, Japan). Before each picture series, a picture of a stage micrometer was photographed to calibrate the measurements which were conducted with the AxioVision LE software (Carl Zeiss Imaging Solutions, München, Germany). The characters examined (Figure 1) were the length of the sporangiophores (n = 25), the height of the first ramification (n = 25), the width of the trunk (n = 25) halfway from the base to the first ramification, the number of branching orders (n = 25), the length of the ultimate branchlets (n = 50 each for the longer and the shorter ultimate branchlet), and the length and the width of the sporangia (n = 100, each). For *Ci. lanatus*, *Cu. maxima*, and *Lu. cylindrica* available measurements were included from Runge & Thines [33], and for *B. dioica*, *C. sativus*, and *S. angulatus* available measurements were included from Runge & Thines [32]. For some characters of *P. cubensis* on *B. dioica*, *C. sativus*, and *S. angulatus* measurements of both studies were available. These were combined as no statistically significant differences were apparent between these. This leads to a duplication of the number of measurements taken for the length of the sporangiophores, the height of the first ramification, and the sporangial dimensions in these hosts. In addition the ratio of sporangiophore length to height of the first ramification, the ratio of the longer to the shorter ultimate branchlet, and the ratio of the length to the width of sporangia were calculated. The data were analysed using the STATISTICA '99 software (StatSoft, Tulsa, OK, USA) applying the Mann-Whitney-U-Test [36] to determine the significance of the differences between the species investigated. The STATISTICA 6.1 software (StatSoft, Tulsa, OK, USA) was used for calculating the Spearman's rank correlation [37] at a significance level of p<0.004 (p<0.05 Bonferroni corrected) to determine a potential correlation of the different characters. Values of the Spearman's rank correlation range from 1 to −1 giving the strength of positive or negative correlations. To evaluate the statistically significant differences regarding the usability in species delimitation the plasticity of each character on each host and among all hosts were determined using the range of the standard deviation ((mean+SD)-(mean−SD)) in relation to the total character variation (maximum-minimum). A classification of the plasticity values was done considering a standard deviation interval of about one third of the total variation interval as moderate variation. Thus a low plasticity is presented by a lower value and a high plasticity by a higher value (host related plasticities: 0.28–0.40 (low), 0.41–0.49 (moderate), 0.50–0.70 (high); overall plasticities: 0.20–0.27 (low), 0.28–0.32 (moderate), 0.33–0.38 (high)). This was done for host related plasticity values altogether and separately for the overall plasticity values. Furthermore, a simple method to estimate the maximum of potentially released infective units per sporangiophore was used in conjunction with the sporulation density to obtain an estimate of the suitability of the hosts for pathogen proliferation. Assuming a perfectly ellipsoid shape, the mean volume of the sporangia was calculated (V = 4/3πab^2^), then divided by the mean volume of one zoospore (V = 5,814 µm^3^) obtained from a previous study with *P. cubensis* and its closest relative *P. humuli* [38], and finally the mean number of zoospores released by one sporangium was multiplied with the potential maximum of ultimate branchlets according to the number of branch orders on the respective host. To estimate the potential infection pressure giving rise to the next asexual cycle, the sporulation density and the infected leaf area was determined. Therefore symptomatic leaves were photographed together with a scale on a desk lighted from below, in order to increase the visibility of infected leaf areas. Areas with sporulation were confirmed and investigated using an Olympus SK60 stereo microscope (Olympus, Hamburg, Germany) with a KL1500 illumination unit (Schott, Mainz, Germany) and afterwards marked on the pictures. From three different leaves of each host, all sporangiophores of one sporulating area with a diameter of 2 mm (3.14 mm^2^) were picked with precision tweezers and counted using a light microscope. Additionally the leaf area and the infected leaf area of the three leaves each were measured using the AxioVision LE software. Afterwards, the potential number of sporangia and zoospores related to the leaf area infected and the total leaf area were calculated.

## Results

Four to nine days after inoculation sporulation could be observed (*Cucumis melo*, *Cyclanthera pedata*, *Sicyos angulatus*, *Thladiantha dubia*: 4d; *Cucumis sativus*, *Cucurbita maxima*, *Lagenaria siceraria*: 5d; *Cucumis anguria*, *Luffa cylindrica*: 6d; *Bryonia dioica*: 7d; *Citrullus lanatus*: 8d; *Cucurbita moschata*: 9d). To the best of our knowledge this is the first report of successful inoculation of *Cy. pedata* and *T. dubia* with *Pseudoperonospora cubensis*. The sporulation density on *Cu. moschata* was very low, therefore, only limited measurements could be done (sporangiophores - length, height of first ramification: n = 18; trunk: n = 19; orders of branching: n = 20; ultimate branchlets: n = 37, each for the longer and the shorter ultimate branchlet; sporangia: n = 25).

The morphological characterisation of *P. cubensis* from the different hosts is given in Table 1, the plasticity values of each character of *P. cubensis* from the different hosts and of the hosts altogether and the classification of the plasticity values are given in Table 2. Since twelve hosts of *P. cubensis* were successfully infected 66 pairwise host-host comparisons were available for statistical analysis.

**Table 1:** Morphological characteristics of *Pseudoperonospora cubensis* on various Cucurbitaceae.

Host species	*Thladiantha dubia*	*Bryonia dioica*	*Luffa cylindrica*
Sporangiophores			
Length	(171–)230–333–436(–649) µm	(198–)255–340–424(–530) µm^c^	(253–)328–393–458(–500) µm^a^
Height of first branching	(107–)183–268–353(–542) µm	(130–)186–252–317(–394) µm^c^	(191–)234–284–335(–372) µm
Ratio length/height of first branching	(1.09–)1.14–1.25–1.37(–1.60)	(1.17–)1.23–1.36–1.49(–1.92)^c^	(1.22–)1.29–1.39–1.49(–1.69)
Width of Trunk	(2.1–)2.9–3.8–4.7(–5.9) µm	(3.6–)4.4–5.4–6.4(–7.5) µm^b^	(3.6–)4.4–5.4–6.4(–7.5) µm
Number of branch orders	(2.00–)3.71–4.48–5.25(–6.00)	(3.00–)3.79–4.56–5.33(–6.00)	(4.00–)5.04–5.80–6.56(–7.00)
Ultimate branchlets			
Length	(2.1–)4.0–7.0–10.1(–18.1) µm	(4.9–)6.5–9.5–12.4(–18.0) µm	(2.1–)5.6–8.2–10.7(–14.7) µm^a^
Length of longer ultimate branchlet	(3.0–)4.7–7.8–11.0(–18.1) µm	(5.6–)7.4–10.4–13.4(–18.0) µm^b^	(4.1–)6.6–9.1–11.5(–14.7) µm
Length of shorter ultimate branchlet	(2.1–)3.4–6.3–9.1(–14.2) µm	(4.9–)6.0–8.5–11.0(–14.5) µm^b^	(2.1–)5.0–7.3–9.6(–12.4) µm
Ratio longer/shorter ultimate branchlet	(1.00–)1.03–1.31–1.58(–2.50)	(1.00–)1.06–1.24–1.41(–1.89)^b^	(1.01–)1.01–1.29–1.60(–2.81)
Sporangia			
Length	(12.1–)18.8–21.9–25.1(–28.2) µm	(17.9–)21.1–23.8–26.6(–33.8) µm^c^	(16.3–)19.1–21.7–24.3(–30.1) µm^a^
Width	(10.6–)13.8–16.0–18.2(–23.2) µm	(11.6–)14.1–15.7–17.3(–21.5) µm^c^	(12.2–)14.6–16.5–18.4(–21.4) µm^a^
Ratio length/width	(1.01–)1.21–1.38–1.54(–1.80)	(1.25–)1.40–1.52–1.65(–1.95)^c^	(1.03–)1.20–1.32–1.44(–1.68)^a^

Host species	*Sicyos angulatus*	*Cyclanthera pedata*	*Cucurbita maxima*
Sporangiophores			
Length	(214–)298–392–486(–584) µm^c^	(185–)220–300–380(–497) µm	(205–)236–289–341(–425) µm^a^
Height of first branching	(155–)216–289–363(–495) µm^c^	(125–)151–209–267(–364) µm	(125–)159–208–257(–372) µm
Ratio length/height of first branching	(1.07–)1.24–1.36–1.49(–1.67)^c^	(1.12–)1.31–1.45–1.58(–1.72)	(1.14–)1.21–1.42–1.63(–1.95)
Width of Trunk	(2.9–)3.3–4.0–4.7(–5.3) µm^b^	(3.7–)3.9–4.9–5.8(–6.7) µm	(3.1–)3.8–4.8–5.8(–7.1) µm
Number of branch orders	(4.00–)4.79–5.56–6.33(–7.00)	(4.00–)4.54–5.28–6.02(–7.00)	(3.00–)3.81–4.52–5.23(–6.00)
Ultimate branchlets			
Length	(2.5–)5.3–8.7–12.(–18.7) µm	(3.1–)5.3–7.4–9.6(–13.7) µm	(3.1–)5.8–8.3–10.9(–16.2) µm^a^
Length of longer ultimate branchlet	(4.0–)6.3–9.7–13.2(–18.7) µm^b^	(4.3–)6.1–8.2–10.3(–13.7) µm	(5.0–)6.6–9.0–11.4(–16.2) µm
Length of shorter ultimate branchlet	(2.5–)4.6–7.8–10.9(–17.1) µm^b^	(3.1–)4.8–6.7–8.5(–11.5) µm	(3.1–)5.2–7.7–10.1(–14.3) µm
Ratio longer/shorter ultimate branchlet	(1.01–)1.04–1.30–1.56(–2.11)^b^	(1.01–)1.08–1.26–1.43(–1.76)	(1.00–)1.01–1.21–1.41(–1.89)
Sporangia			
Length	(12.0–)17.9–20.8–23.7(–30.5) µm^c^	(9.3–)17.0–19.6–22.2(–25.5) µm	(13.6–)20.0–23.4–26.8(–32.9) µm^a^
Width	(9.78–)12.5–14.4–16.2(–19.2) µm^c^	(8.6–)12.5–14.1–15.7(–19.6) µm	(11.7–)14.0–16.0–17.9(–23.4) µm^a^
Ratio length/width	(1.01–)1.31–1.45–1.60(–1.80)^c^	(1.05–)1.27–1.39–1.51(–1.67)	(1.01–)1.29–1.47–1.65(–1.84)^a^

Host species	*Cucurbita moschata*	*Citrullus lanatus*	*Lagenaria siceraria*
Sporangiophores			
Length	(173–)195–266–337(–422) µm	(153–)225–301–375(–397) µm^a^	(230–)277–345–413(–498) µm
Height of first branching	(69.4–)130–181–232(–264) µm	(108–)163–228–293(–317) µm	(162–)208–263–318(–372) µm
Ratio length/height of first branching	(1.26–)1.14–1.52–1.90(–2.84)	(1.16–)1.22–1.34–1.45(–1.62)	(1.18–)1.25–1.32–1.39(–1.49)
Width of Trunk	(3.3–)4.1–4.8–5.5(–6.3)µm	(2.9–)3.5–4.4–5.4(–6.1) µm	(3.3–)4.1–4.9–5.7(–6.6) µm
Number of branch orders	(3.00–)3.56–4.45–5.34(–6.00)	(3.00–)3.79–4.56–5.33(–6.00)	(4.00–)3.94–4.64–5.34(–6.00)
Ultimate branchlets			
Length	(3.3–)5.7–9.1–12.5(–17.2) µm	(3.6–)5.5–9.2–12.8(–18.4) µm^a^	(2.2–)5.1–8.1–11.1(–15.7) µm
Length of longer ultimate branchlet	(4.4–)6.2–9.8–13.4(–17.2) µm	(5.1–)6.7–10.5–14.2(–18.4) µm	(5.5–)6.6–9.4–12.2(–15.7) µm
Length of shorter ultimate branchlet	(3.3–)5.4–8.4–11.5(–15.6) µm	(3.6–)4.8–7.9–11.0(–14.9) µm	(2.2–)4.2–6.8–9.3(–12.5) µm
Ratio longer/shorter ultimate branchlet	(1.00–)1.00–1.17–1.34(–1.65)	(1.01–)1.07–1.36–1.65(–2.44)	(1.04–)1.16–1.46–1.75(–2.58)
Sporangia			
Length	(17.8–)19.8–22.3–24.8(–29.7) µm	(14.3–)19.1–21.6–24.1(–27.1) µm^a^	(18.0–)20.4–23.4–26.5(–38.8) µm
Width	(12.6–)13.4–14.9–16.4(–19.0) µm	(10.0–)13.4–15.5–17.5(–19.7) µm^a^	(13.9–)15.6–17.7–19.9(–27.5) µm
Ratio length/width	(1.29–)1.37–1.50–1.63(–1.76)	(1.01–)1.25–1.41–1.57(–1.88)^a^	(1.03–)1.19–1.33–1.46(–1.66)

Host species	*Cucumis anguria*	*Cucumis sativus*	*Cucumis melo*	Overall mean	Maximum deviation
Sporangiophores					
Length	(251–)273–351–429(–498) µm	(173–)259–354–449(–606) µm^c^	(267–)354–452–550(–642) µm	349	30%
Height of first branching	(123–)175–229–283(–308) µm	(101–)182–257–332(–434) µm^c^	(105–)246–327–409(–472) µm	255	30%
Ratio length/height of first branching	(1.33–)1.30–1.56–1.82(–2.62)	(1.16–)1.25–1.40–1.54(–1.91)^c^	(1.13–)1.17–1.41–1.66(–2.53)	1.39	13%
Width of Trunk	(4.8–)5.6–6.8–8.0(–9.5) µm	(3.1–)4.3–5.3–6.4(–7.2) µm^b^	(3.2–)4.2–4.9–5.6(–6.3) µm	5.0	37%
Number of branch orders	(4.00–)5.22–5.92–6.62(–7.00)	(4.00–)4.60–5.36–6.12(–7.00)	(4.00–)4.35–4.96–5.57(–6.00)	5.0	18%
Ultimate branchlets					
Length	(3.8–)7.0–9.8–12.5(–20.3) µm	(2.9–)5.6–8.4–11.1(–15.2) µm	(3.4–)6.4–9.9–13.4(–21.4) µm	8.6	19%
Length of longer ultimate branchlet	(5.4–)8.1–10.8–13.6(–20.3) µm	(4.0–)6.6–9.4–12.1(–15.2) µm^b^	(4.5–)7.3–10.9–14.5(–21.4) µm	9.6	19%
Length of shorter ultimate branchlet	(3.8–)6.3–8.7–11.1(–14.6) µm	(2.9–)4.9–7.3–9.8(–13.7) µm^b^	(3.4–)5.6–8.9–12.1(–19.5) µm	7.6	19%
Ratio longer/shorter ultimate branchlet	(1.03–)1.09–1.26–1.42(–1.64)	(1.01–)1.08–1.32–1.56(–2.23)^b^	(1.02–)1.06–1.26–1.45(–1.92)	1.29	14%
Sporangia					
Length	(16.9–)20.8–23.6–26.4(–32.2) µm	(14.2–)19.2–22.8–26.4(–36.9) µm^c^	(10.2–)18.7–21.2–23.7(–27.8) µm	22.2	12%
Width	(12.1–)14.7–16.5–18.3(–22.1) µm	(10.3–)14.1–16.3–18.5(–25.1) µm^c^	(9.9–)13.4–14.8–16.1(–18.6) µm	15.7	13%
Ratio length/width	(1.20–)1.33–1.43–1.54(–1.65)	(1.00–)1.26–1.40–1.54(–1.78)^c^	(1.03–)1.31–1.44–1.56(–1.69) µm	1.42	8%

All measurements given in the form (minimum-) standard deviation towards the minimum - mean - standard deviation towards the maximum (-maximum).

a Runge & Thines [33].

b Runge & Thines [32].

c Runge & Thines [32], [33] combined.

**Table 2:** Plasticity values of *Pseudoperonospora cubensis* on various Cucurbitaceae.

Host species	*Thladiantha dubia*	*Bryonia dioica*	*Luffa cylindrica*	*Sicyos angulatus*	*Cyclanthera pedata*
Sporangiophores					
Length	0.43	0.51	0.53	0.51	0.51
Height of first branching	0.39	0.50	0.56	0.43	0.48
Ratio length/height of first branching	0.45	0.34	0.43	0.41	0.45
Width of Trunk	0.47	0.52	0.50	0.60	0.61
Number of branch orders	0.39	0.51	0.51	0.51	0.49
Ultimate branchlets					
Length	0.38	0.45	0.40	0.42	0.40
Length of longer ultimate branchlet	0.41	0.49	0.46	0.46	0.45
Length of shorter ultimate branchlet	0.47	0.52	0.45	0.43	0.45
Ratio longer/shorter ultimate branchlet	0.37	0.40	0.34	0.47	0.47
Sporangia					
Length	0.39	0.35	0.38	0.32	0.32
Width	0.34	0.31	0.41	0.39	0.29
Ratio length/width	0.41	0.36	0.37	0.36	0.38

Host species	*Cucurbita maxima*	*Cucurbita moschata*	*Citrullus lanatus*	*Lagenaria siceraria*
Sporangiophores				
Length	0.48	0.57	0.62	0.51
Height of first branching	0.40	0.53	0.62	0.53
Ratio length/height of first branching	0.52	0.48	0.50	0.46
Width of Trunk	0.52	0.48	0.60	0.48
Number of branch orders	0.48	0.59	0.51	0.70
Ultimate branchlets				
Length	0.38	0.49	0.49	0.44
Length of longer ultimate branchlet	0.43	0.56	0.56	0.55
Length of shorter ultimate branchlet	0.44	0.50	0.55	0.50
Ratio longer/shorter ultimate branchlet	0.46	0.52	0.41	0.38
Sporangia				
Length	0.35	0.42	0.39	0.29
Width	0.33	0.47	0.43	0.32
Ratio length/width	0.44	0.53	0.37	0.43

Host species	*Cucumis anguria*	*Cucumis sativus*	*Cucumis melo*	Mean	Overall mean
Sporangiophores					
Length	0.63	0.44	0.52	0.52	0.38
Height of first branching	0.58	0.45	0.44	0.49	0.32
Ratio length/height of first branching	0.40	0.40	0.35	0.43	0.20
Width of Trunk	0.53	0.53	0.45	0.52	0.32
Number of branch orders	0.47	0.50	0.61	0.52	0.36
Ultimate branchlets					
Length	0.33	0.46	0.39	0.42	0.32
Length of longer ultimate branchlet	0.37	0.49	0.42	0.47	0.34
Length of shorter ultimate branchlet	0.45	0.46	0.40	0.47	0.32
Ratio longer/shorter ultimate branchlet	0.54	0.39	0.43	0.43	0.27
Sporangia					
Length	0.37	0.32	0.28	0.35	0.22
Width	0.37	0.30	0.31	0.36	0.23
Ratio length/width	0.46	0.36	0.38	0.40	0.32

Plasticity categories; host related plasticities: 0.28–0.40 (low), 0.41–0.49 (moderate), 0.50–0.70 (high); overall plasticities: 0.20–0.27 (low), 0.28–0.32 (moderate), 0.33–0.38 (high). The classification of the plasticity values was done considering a standard deviation interval of about one third of the total variation interval as moderate variation. A low plasticity is presented by a lower value and a high plasticity by a higher value.

**Figure 2:**
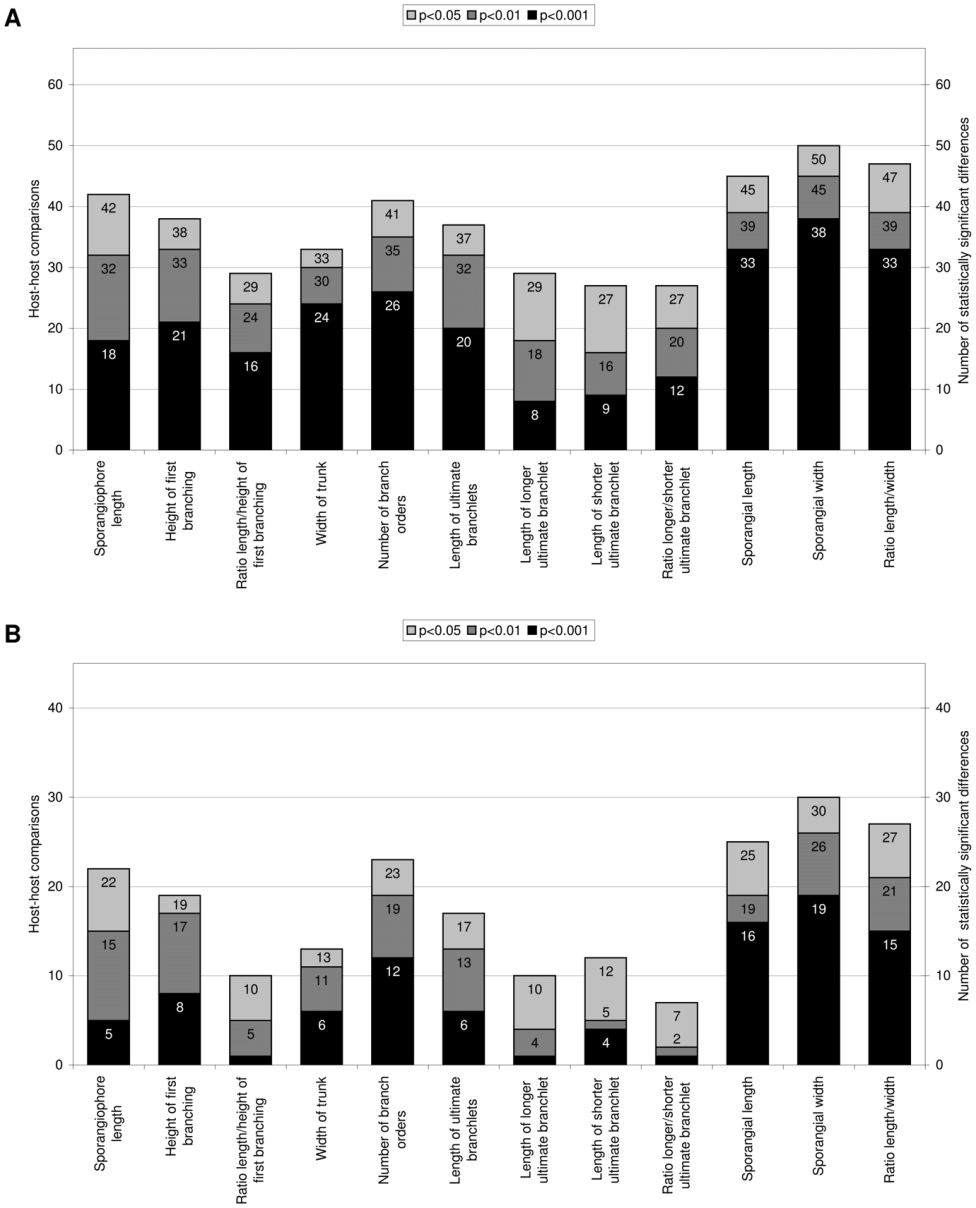
Number of statistically significant differences in morphological characteristics of *Pseudoperonospora cubensis* on a set of twelve cucurbitaceous hosts for all 66 possible host-host comparisons (A) and without the respectively two most deviating hosts, resulting in 45 possible host-host comparisons (B). Excluded in B were the hosts as follows for the characteristics from left to right: 1. *Cucumis melo*, *Luffa cylindrica*; 2. *C. melo*, *Cucurbita moschata*; 3. and 4. *Thladiantha dubia*, *Cucumis anguria*; 5. *C. anguria*, *Lu. cylindrica*; 6. and 7. *T. dubia*, *Cyclanthera pedata*; 8. *T. dubia*, *C. anguria*; 9. *Lagenaria siceraria*, *Cu. moschata*; 10. *Cy. pedata*, *Sicyos angulatus*; 11. *La. siceraria*, *Cy. pedata*; 12. *Lu. cylindrica*, *La. siceraria*.

The sporangiophores varied in their absolute lengths from 153 µm on *Ci. lanatus* to 649 µm on *T. dubia*, in their mean lengths from 266 µm on *Cu. moschata* to 452 µm on *C. melo*, and in their plasticity from 0.43 on *T. dubia* to 0.63 on *C. anguria* (mean 0.52). The height of the first ramification varied in its extremes from 69.4 µm on *Cu. moschata* to 542 µm on *T. dubia*, the mean of the height of the first ramification from 181 µm on *Cu. moschata* to 327 µm on *C. melo*, and its plasticity from 0.39 on *T. dubia* to 0.62 on *Ci. lanatus* (mean 0.49). The lowest ratio of the length of the sporangiophores to the height of the first ramification was observed in *T. dubia* (mean 1.25), the highest in *C. anguria* (mean 1.56, mean of plasticity values 0.43). Moreover the length of the sporangiophores and the height of the first ramification were positively correlated on all hosts with a correlation index of 0.888 (all correlation values significant at a significance level of at least p<0.004). The length of the sporangiophores varied significantly (p<0.05) in 42 of the 66 host-host comparisons (p<0.01: 32; p<0.001: 18; Figure 2A) with the highest overall plasticity value of 0.38. In contrast, the ratio of the length to the height of the first ramification varied significantly only in 29 (24, 16) comparisons and had the lowest overall plasticity with 0.20. After eliminating the two most diverging hosts from each of the comparisons, the length of the sporangiophores showed 22 (15, 5) significant differences (*C. melo* and *Lu. cylindrica* were eliminated; Figure 2B), and the ratio of the length to the height of the first ramification showed only 10 (5, 1) significant differences (*T. dubia* and *C. anguria* were eliminated). Although *Ci. lanatus*, *Cu. maxima*, and *T. dubia* showed positive correlations between the length of the sporangiophores and the width of the trunk, no correlation was observed between these characters considering all hosts. The width of the trunk varied from 2.1 µm on *T. dubia* to 9.5 µm on *C. anguria*, the mean values varied from 3.8 µm to 6.8 µm on the same hosts, and the plasticity varied from 0.45 on *C. melo* to 0.61 on *Cy. pedata* (mean 0.52). The trunk showed 33 (30, 24) significant differences among the 66 host-host comparisons with an overall plasticity of 0.32, and after elimination of the two extremes, *T. dubia* and *C. anguria*, only 13 (11, 6) significant differences were found. In the morphology of the sporangiophores including the number of branching orders *B. dioica* had no statistically significant difference to *La. siceraria*, but one difference to *Ci. lanatus* and *C. sativus*, respectively. *Ci. lanatus* was not different from *La. siceraria* and *Cu. maxima*, whereas *Cu. maxima* had no differences compared to *Cu. moschata*. Both were in differing in one character from *Cy. pedata*. Also *Lu. cylindrica* and *S. angulatus* had only one statistically significant difference. The number of branching orders ranged from two to seven with the mean values ranging from 4.45 on *Cu. moschata* to 5.92 on *C. anguria*, and plasticity values from 0.39 on *T. dubia* to 0.70 *La. siceraria* (mean: 0.52). The number of branching orders showed 41 (35, 26) significant differences and a high overall plasticity of 0.36. After elimination of *C. anguria* and *Lu. cylindrica* there were still 23 (19, 12) significant differences among the remaining 45 host-host comparisons left. Considering all hosts there was a positive correlation between the length of the sporangiophores and the number of branch orders with a correlation index of 0.722.

The overall length of all ultimate branchlets ranged from 2.1 µm on *Lu. cylindrica* and *T. dubia* to 21.4 µm on *C. melo*. The mean values of the ultimate branchlets ranged from 7.0 µm (mean of longer ultimate branchlets 7.8 µm; mean of shorter ultimate branchlets 6.3 µm; mean of plasticity values: 0.47 each) on *T. dubia* to 9.9 µm (10.9 µm, 8.9 µm) on *C. melo*, and the plasticity ranged from 0.33 on *C. anguria* to 0.49 on *Ci. lanatus* and *Cu. moschata* (mean 0.42). The lowest ratio of the longer to the shorter ultimate branchlet had *P. cubensis* on *Cu. moschata* (mean 1.17), whereas on *La. siceraria* it had the highest ratio (mean 1.46). *Pseudoperonospora cubensis* on *Lu. cylindrica* had the lowest plasticity (0.34) and the highest (0.54) was observed on *C. anguria* (mean 0.43).

Considering the longer ultimate branchlets separately 29 (18, 8) host-host comparisons were significantly different, with *T. dubia* and *Cy. pedata* being the most diverging hosts. The shorter ultimate branchlets showed 27 (16, 9) statistically significant differences. Also the ratio of the longer to the shorter ultimate branchlets showed 27 (20, 12) differences, but after *La. siceraria* and *Cu. moschata* were eliminated, only 7 (2, 1) differences were left. The ultimate branchlets had a high overall plasticity of 0.32 (longer 0.34; shorter 0.32). However, the ratio of the longer to the shorter ultimate branchlets had a low overall plasticity of 0.27.

On all hosts a positive correlation of the length of the longer to the length of the shorter ultimate branchlet and a negative correlation of the length of the shorter ultimate branchlet to the ratio of the dichotomous ultimate branchlets were observed. No correlation of the length of the longer to the ratio of the dichotomous branchlets could be found, indicating that the shorter ultimate branchlet is the determining factor for the ratio of the length of the longer to the shorter ultimate branchlet. Among all hosts a very strong correlation (0.916) between the lengths of the dichotomous ultimate branchlets was found.

In the characteristics of the ultimate branchlets, *P. cubensis* on *Lu. cylindrica* had no statistically significant differences compared to *Ci. lanatus*, *C. sativus*, *Cu. maxima*, and *S. angulatus*. Furthermore *Cu. maxima* was not different from *Cu. moschata*, and *Ci. lanatus* was not different from *C. anguria* and *C. melo*. These two were neither different from each other nor from *B. dioica*, which had only one significant difference when compared to *Ci. lanatus*. *Cucurbita moschata* had only one significantly different combination to each of the above mentioned hosts. Furthermore *La. siceraria* had only one significant difference when compared to *Cu. maxima*, *Lu. cylindrica*, and *S. angulatus*.

The sporangia ranged in length from 9.3 µm on *Cy. pedata* to 38.8 µm on *La. siceraria* and in width from 8.6 µm to 27.5 µm on the same hosts, while the mean values ranged from 19.6 µm on *Cy. pedata* to 23.8 µm on *B. dioica* in length and from 14.1 µm on *Cy. pedata* to 17.7 µm on *La. siceraria* in width. The lowest ratio of length to width of sporangia was observed for *Lu. cylindrica* (mean 1.32) and the highest ratio had *B. dioica* (mean 1.52). The plasticity of the sporangial length ranged from 0.28 on *C. melo* to 0.42 on *Cu. moschata* (mean 0.35) and the plasticity of the sporangial width ranged from 0.29 on *Cy. pedata* to 0.47 on *Cu. moschata* (mean 0.36), while the plasticity of the ratio of length to width ranged from 0.36 on *B. dioica*, *C. sativus*, and *S. angulatus* to 0.53 on *Cu. moschata* (mean 0.40).

Concerning the length of the sporangia, 45 (39, 33) of the 66 host-host comparisons were statistically significant different. For the width of sporangia, 50 (45, 38) of the combinations were different. For the ratio of sporangial length to width, there were 47 (39, 33) differences found. However, only the overall plasticity of the ratio of sporangial length to width was high with 0.32. The overall plasticity of the sporangial length and the sporangial width was very low, with 0.22 and 0.23, respectively.

On all hosts there was a positive correlation of the length to the width of the sporangia, and also of the length to the ratio of length to width. Furthermore, on almost all hosts, except for *C. melo*, *Cu. moschata*, and *Cy. pedata*, the width of sporangia was correlated negative to the ratio of length to width. Considering all hosts there was a positive correlation (0.664) of the length to the width of the sporangia. *T. dubia* had no significant differences to *C. sativus* and *Ci. lanatus*. In addition *C. melo* was not significantly different from *Cu. moschata*. But all other comparisons had at least one statistically significant difference.

**Figure 3:**
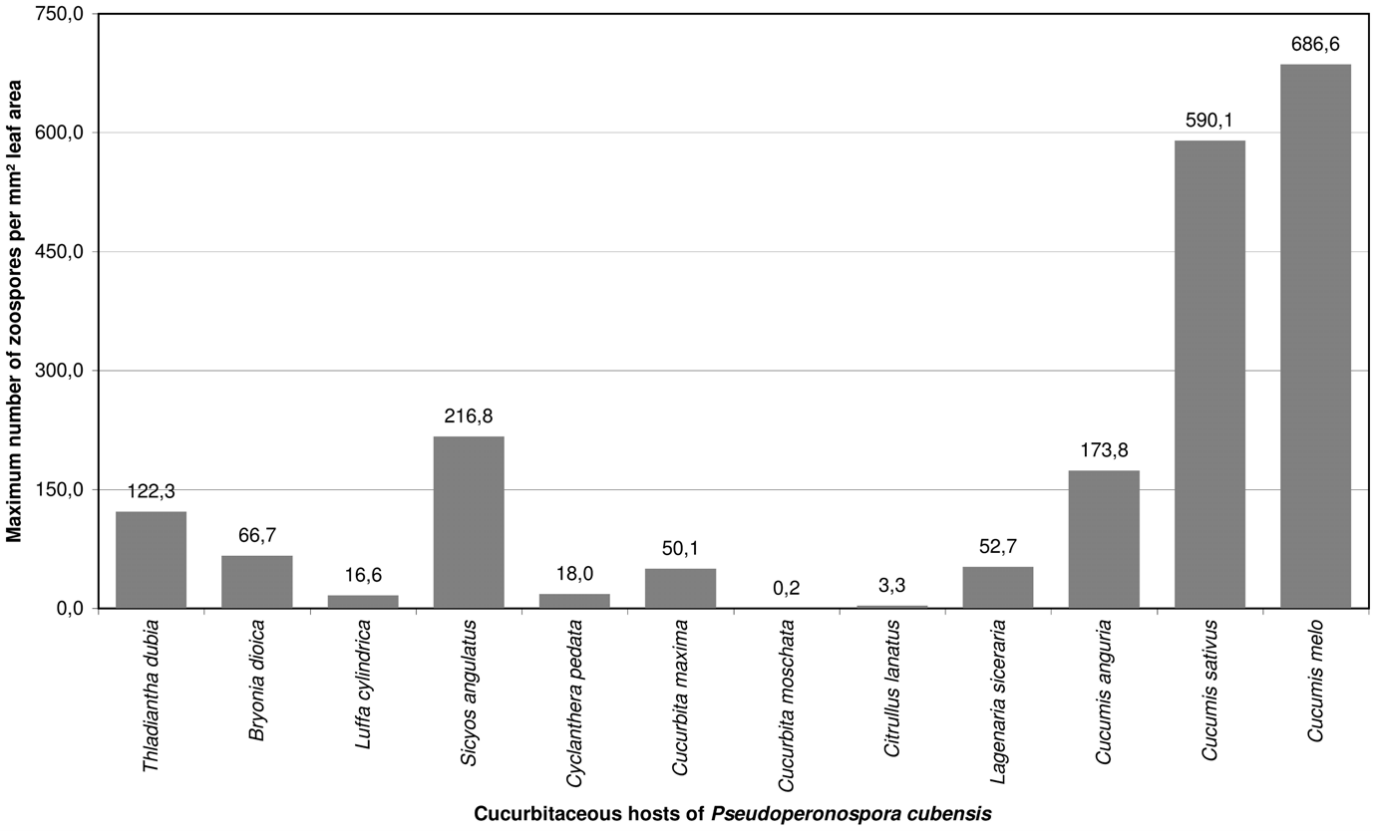
Estimation of the total amount of infective units of *Pseudoperonospora cubensis* per leaf area of the respective cucurbitaceous hosts.

Regarding the sporangial volume, *P. cubensis* on *Cy. pedata* had the smallest sporangia with a mean volume of 2.045 µm^3^ (Table 3), and *La. siceraria* had the biggest sporangia with a mean volume of 3.854 µm^3^ (*C. sativus*: mean 3,165 µm^3^).Thus, considering the calculated mean of branch orders of the respective host the potential maximum number of released zoospores of one sporangiophore ranges from 98 on *Cu. moschata* to 350 on *C. anguria* (*C. sativus*: 224). The number of sporangiophores covering an infected leaf area of 1 mm^2^ ranges in the mean from 5.5 on *Lu. cylindrica* to 57.3 on *S. angulatus* (*C. sativus*: 21.4; for *Cu. moschata* was excluded because of too sparse sporulation). Considering the infected leaf area in relation to the whole leaf surface, the number of sporangiophores per 1 cm^2^ ranges from 3.0 on *Ci. lanatus*, where only 0.3% of the surface shows sporulation, to 529.4 on *C. melo*, where 22.7% of the surface shows sporulation (*C. sativus* 263.3, 12.3%). This leads to a potential mean number of 0.7 sporangia and 3.3 zoospores per mm^2^ leaf area on *Ci. lanatus* and to a potential number of 164.8 sporangia and 686.6 zoospores on *C. melo* (*C. sativus* 108.2, 590.1). Considering that all sporangiophores were picked from the leaf surface in *Cucurbita moschata*, this host had only 0.2 sporangiophores per 1 cm^2^ of leaf area and therefore only a potential maximum number of zoospores of 0.2 per mm^2^. A comparison of the potential zoospore discharge is given in Figure 3.

**Table 3:** Sporulation density and potential numbers of sporangia and zoospores.

Host species	*Thladiantha dubia*	*Bryonia dioica*	*Luffa cylindrica*	*Sicyos angulatus*
Leaf area in mm^2^	1,897	1,718	6,176	6,000
Sporulating leaf area in mm^2^	113	75	62	125
Sporulating leaf area in % of overall leaf area	6.0	4.4	1.0	2.1
Sporangiophores per mm^2^ sporulating leaf area	18.0	12.2	5.5	57.3
Sporangiophores per **cm^2^** leaf area	107.9	53.4	5.6	119.0
Theoretical approach				
Max. number of sporangia/sporangiophore	22	24	56	47
Volume of sporangia in µm^3^ (if perfect ellipsoid)	2,945	3,072	3,111	2,239
Max. number of released zoospores per sporangiophore	113	125	298	182
Max. number of sporangia per mm^2^ leaf area	24.1	12.6	3.1	56.2
Max. number of zoospores per mm^2^ leaf area	122.3	66.7	16.6	216.8

Host species	*Cyclanthera pedata*	*Cucurbita maxima*	*Cucurbita moschata*	*Citrullus lanatus*
Leaf area in mm^2^	4,030	10,097	8,337	4,316
Sporulating leaf area in mm^2^	45	645	1	14
Sporulating leaf area in % of overall leaf area	1.1	6.4	0.0	0.3
Sporangiophores per mm^2^ sporulating leaf area	11.9	6.4	20.0	9.5
Sporangiophores per **cm^2^** leaf area	13.2	40.6	0.2	3.0
Theoretical approach				
Max. number of sporangia/sporangiophore	39	23	22	24
Volume of sporangia in µm^3^ (if perfect ellipsoid)	2,045	3,116	2,604	2,704
Max. number of released zoospores per sporangiophore	137	123	98	110
Max. number of sporangia per mm^2^ leaf area	5.1	9.3	0.0	0.7
Max. number of zoospores per mm^2^ leaf area	18.0	50.1	0.2	3.3

Host species	*Lagenaria siceraria*	*Cucumis anguria*	*Cucumis sativus*	*Cucumis melo*
Leaf area in mm^2^	9,067	8,181	7,805	7,734
Sporulating leaf area in mm^2^	453	462	959	1,754
Sporulating leaf area in % of overall leaf area	5.0	5.6	12.3	22.7
Sporangiophores per mm^2^ sporulating leaf area	6.4	8.8	21.4	23.3
Sporangiophores per **cm^2^** leaf area	31.8	49.7	263.3	529.4
Theoretical approach				
Max. number of sporangia/sporangiophore	25	61	41	31
Volume of sporangia in µm^3^ (if perfect ellipsoid)	3,854	3,350	3,165	2,417
Max. number of released zoospores per sporangiophore	165	349	224	129
Max. number of sporangia per mm^2^ leaf area	7.9	30.1	108.2	164.8
Max. number of zoospores per mm^2^ leaf area	52.7	173.8	590.1	686.6

## Discussion

Sporulation was first observed on all hosts 4–9 days following inoculation with *Pseudoperonospora cubensis*. An incubation period of 4–12 days has been previously reported, depending on environmental conditions, inoculum load [39], and resistance or susceptibility of the host plant [40]. The variability in the occurrence of first sporulation is in line with previous results from investigations into the genetic variation in resistance to *P. cubensis* among cucurbits [41], [42]. The susceptibility of the different cucurbitaceous hosts can be evaluated on the basis of the onset of the sporulation, and also the density of the sporulation. Sporulation on *Cucumis sativus* had the highest density, on *Sicyos angulatus* there were also leaf areas with extremely dense sporulation, but sporulation intensity on *Luffa cylindrica* or *Cyclanthera pedata* was rather low, although in the latter host sporulation occurred already after 4 days. *Cucurbita moschata* was almost resistant to *P. cubensis*, with late onset of sporulation and few emerging sporangiophores. Pathotype specificity was described previously for *Cu. moschata*, but the genetics of host resistance to *P. cubensis* is currently unknown [43]. Nevertheless, if infection experiments with other strains yield similar results, *Cu. moschata* might be a good resource for resistance genes against *P. cubensis*.

The Cucurbitaceae family includes more than 118 genera with 825 species [44]. Of these 20 genera and ca. 60 species are known to be hosts of *P. cubensis* [45], [46]. We have found two new potential hosts, *Cy. pedata* and *Thladiantha dubia*, which were susceptible under laboratory conditions. Considering the infection potential of *P. cubensis* and the ongoing discovery of new pathotypes, it seems likely that more cucurbit species will be reported as hosts in the future.

Previous reports of a variety of *Pseudoperonospora* species specific to different cucurbitaceous hosts [22] could not be confirmed in molecular phylogenetic investigations [20], [21]. However, two genetically distinct, but morphologically cryptic lineages were found by Runge et al. [21]. Before the onset of molecular phylogenetic investigations, downy mildew species were mainly described on the basis of the host matrix and minor morphological differences on similar hosts, also in *Pseudoperonospora* (e.g. [22]). Although it has been shown that the host matrix has a significant impact on the morphology of some characteristics of *Pseudoperonospora* [33], the extent of this phenomenon could not be fully described previously. Here, we demonstrate that the length of the sporangiophores is a highly variable character depending on the host matrix. It ranges from 153 µm on *Ci. lanatus* to 649 µm on *T. dubia* and has a very high overall plasticity of 0.38. In addition the maximum character deviation of 30% from the overall mean of 349 µm raise doubts if this character is suitable for species delimitation. Although the measured sporangiophores of previous studies [20], [23], [47]–[49] were comparatively smaller, they also showed a high variability. Measurements of *P. cubensis* on *La. siceraria* done by Choi & Shin [48] fit perfectly to our results on *La. siceraria* and measurements on *Cu. moschata* made by Choi et al. [20] were also similar to our results on *Cu. moschata*. Minor differences to other studies may be due to the dependence of sporangiophore morphology on temperature as shown by Iwata [23], or on other environmental conditions, especially if specimens were collected in the wild. At comparable temperatures, sporangiophores of *P. cubensis* from *C. sativus* measured by Iwata [23] were smaller than in the present study but sporangiophores taken from *Cu. moschata* were larger than reported here, at comparable temperatures. Although Iwata explained the observed differences with the existence of different biological species of *P. cubensis* with different pathogenicity, these differences are possibly reflecting the intraspecific variability of *P. cubensis* [31]. We thus used both fixed environmental conditions and the same isolate of *P. cubensis* for all the inoculations. Therefore, the observed differences must be induced by the host matrix [33], i.e. differences in the susceptibility and nutrient supply by the hosts [40]. Modification by differences in plant nutrition [50] should be weak, as all plants in the greenhouse were grown in the same soil, as were the plants cut from outdoors. The sporangiophores were very plastic in both their lengths and the height of the first ramification. This led to a lower amount of significant differences. But with a high total plasticity, these characteristics differed significantly in many host-host comparisons and the mean values on the different hosts deviated by 30% in maximum compared to the overall means (349 µm, 255 µm, respectively). Therefore, sporangiophore length and height of the first ramification are not suitable characters for species delimitation. While the height of the first ramification is a character of high variability and high plasticity (0.32), similar to the sporangiophore length, the ratio of these characters is more stable, also shown by the lowest overall plasticity (0.20) and the relatively low amount of statistically significant differences, especially if the most divergent hosts are ignored. Notably the ratio on *C. sativus* matches the value on *C. sativus* in Iwata's [23] analysis, despite the differences in measurements of the respective characteristics of the sporangiophores. Considering the very low overall plasticity (0.20) and a maximum deviation of only 13% from the overall mean (1.39) renders the ratio of sporangiophore length and the height of the first ramification a character potentially useful for species delimitation.

Regarding the width of the trunk, half of all host comparisons were significantly different. In general, the trunk shows rather few statistically supported differences, primarily caused by high to very high plasticity values. The trunk was reported to be slightly thicker in previous studies [23], [47]–[49], but in general the values were comparable. Trunk thickness is a character that is often measured in species descriptions and sometimes is useful for species delimitation [34], but the high overall plasticity in this study and in measurements of *P. humuli* [32], [51], the closest relative to *P. cubensis*, and the maximum deviation of 37% from the overall mean (4.97) indicate that for *Pseudoperonospora* this character is not suitable for differentiating between closely related species.

The order of branching is limited in its variability, as it ranges in most cases from three or four to six or seven. However, the frequency distribution of branch orders makes this character highly variable on different hosts with a very high total plasticity of 0.36. Statistically significant differences were observed for order of branching for 41 of 66 host-host comparisons, despite a high plasticity on almost all hosts. Comparison with data from other authors [23], [47], [48] reveals highly similar figures for the respective hosts, thus suggesting the dependence of the branching orders on the host plant. Thus, although the host related means deviate by a moderate 18% compared to the overall mean (5.02), this character does not seem to be suitable for species delimitation in closely related downy mildews, as the variability of this character is in an overall narrow window. The positive correlation between the length of the sporangiophores and the order of branching indicates a genetic pattern and it seems likely that proper nourishment, caused by adaptation to the host plant, results in longer and more frequently branched sporangiophores carrying more sporangia. Consequently, branching order is also correlated to the amount of possible infections for the next generation.

The ultimate branchlets are a character of moderate variability. Although extremes of 2.1 µm and 21.4 µm were measured, the lengths of the longer and the shorter ultimate branchlets had the fewest statistically significant differences. But the differing plasticity values among the hosts, the high total plasticity and the moderate maximum deviation (19%, each) of the host related means from the overall means (overall 8.6 µm, longer 9.6 µm, shorter 7.6 µm) make it difficult to use these characteristics with confidence in species delimitation. The most stable character is the ratio of the longer to the shorter ultimate branchlet, represented by the fewest statistically significant differences and a low overall plasticity of 0.27 with means deviating by 14% from the overall mean (1.29). This is also expressed by the very strong correlation of the lengths of the longer to the lengths of the shorter ultimate branchlets. In species descriptions, measurements of the ultimate branchlets are often lacking and their ratio has only been considered in a few very recent studies [12], [34]. In case of species that are not sister taxa but closely related, like for *Plasmopara angustiterminalis* and *Plasmopara* sp. from *Ambrosia artemisiifolia* and also the case of *Peronospora swinglei* and *P. belbahrii*, the lengths of the ultimate branchlets as well as their ratio support the molecular phylogenetic segregation of the species [12], [34]. But for very closely related species, as has been shown by Runge & Thines [32] for *P. humuli* on *S. angulatus*, even moderate deviation of these characters could lead to a breakdown of the significance values. Unfortunately there is no information about ultimate branchlets in the description of any *Pseudoperonospora* species (summarised in [26]) for further comparison of this feature. However, we conclude that at least for species that have already moderately diverged, the lengths of the longer and shorter ultimate branchlets and especially their ratio should be considered and might be useful for species delimitation.

Size and shape of the sporangia of *P. cubensis* are highly affected by the host matrix. The length of the sporangia ranges from 9.3 µm on *Cy. pedata* to 38.8 µm on *La. siceraria* (mean 22.2 µm) and the width from 8.6 µm to 27.5 µm (mean 15.7 µm) on the same respective hosts. The ratio of length to width ranges from 1.00 to 1.95 (mean 1.42). The observed variability is in line with previous observations, as Sawada [22] segregated some species from *P. cubensis* on the basis of host matrix and sporangial dimensions. Due to the known high interspecific variability Waterhouse & Brothers [26] and Gäumann [6], [7] also focused their work on sporangial dimensions. Whether genetic background (also due to phylogenetic divergence), host matrix, or environmental conditions could also be responsible for the high variability had never been studied systematically. However, there is an increasing body of evidence from recent studies combining molecular phylogenetics with morphological investigations, e.g. from *Bremia* [9], *Peronospora* [8], [12], [52], [53], and *Hyaloperonospora* [54], [55] that sporangial dimensions might be a useful character for species delimitation. In our investigation, there was a positive correlation between the length and the width of sporangia, so the tendency for longer sporangia to also be broader is given. Statistically significant differences were observed for length of sporangia in 45, for width of sporangia in 50, and for the ratio of length to width in 47 of 66 host-host comparisons. In all three characters, at least 33 comparisons had the highest significance level (p<0.001), despite the fact that a single isolate was used and all environmental conditions were controlled. The sporangia were statistically not significantly different only from *T. dubia* and *C. sativus*, *T. dubia* and *Ci. lanatus*, as well as *C. melo* and *Cu. moschata*. The high amount of significant differences is caused by the low plasticity values on the various hosts. Overall, the very low total plasticitiy values of the sporangial length (0.22) and the sporangial width (0.23) make these characteristics very stable and therefore suitable for species delimitations. In contrast, the ratio of these characteristics has a much higher plasticity of 0.32, but under controlled conditions, our study shows that in *P. cubensis* the ratio of the length and the width of the sporangia can vary by about 8%. Given the broad range of sporangial shapes, this character might nonetheless be suitable for species delimitation, decided on a case for case basis, if the intraspecific variation is taken into consideration. For the included characteristics, there was not a single combination without statistically significant differences, highlighting the strong influence of the host matrix on the morphology of downy mildews [33]. Taking into account that the variability under natural environmental conditions will be even high due to the dependence of the morphology on temperature, humidity, and light [23], [27], [28], care has to be taken when species are delimited solely on the basis of morphology. Considering the possible variability of the pathogen taken from different parts of the same plant [29] and the influence of the host matrix [26], [33], the importance of molecular phylogenetic investigations in addition to morphological investigations becomes obvious. In the absence of molecular data, species delimitation is uncertain, especially if hosts are closely related and morphological differences are subtle. Morphologically indistinguishable pathogens might, however, be different species [4], [21], but the same pathogen may also look significantly different depending on the influence of diverse factors given above, especially when considering the potentially strong influence of the host matrix on morphological characteristics.

However, this study reveals that the ratio of the height of the first ramification and sporangiophore length, ratio of the longer to the shorter ultimate branchlets, and especially the length and width of sporangia, as well as, with some reservations, their ratio, are the most suitable characteristics for species delimitations in *Pseudoperonospora*. Additional investigations of intraspecific variation and the influence of host matrix on other downy mildew genera could be useful to investigate if the findings in this study can be generalised for downy mildews.

In a previous study [33], it appeared that the morphological differences of *P. cubensis* might correlate with the phylogenetic position of their hosts in the Cucurbitaceae. In the light of the enlarged dataset in this study and considering the current phylogeny of the Cucurbitaceae [56], [57], we found that pathogen morphology on phylogenetically closely related species tended to be similar. For instance *Cu. maxima* and *Cu. moschata* of the Cucurbiteae, *Ci. lanatus* and *La. siceraria* of the Benincaseae, and *Lu. cylindrica* and *S. angulatus* of the Sicyoeae were similar to each other, even in highly variable characters, like the length of the sporangiophores and the number of branch orders, but more dissimilar to other tribes. In addition, on some of the host species (e.g. *Cy. pedata* and *Ci. lanatus*) some characters were similar to the neighbouring tribe, thus occupying an intermediate position between the neighbouring tribe and the tribe of the host species. But, this correlation is not apparent considering the more basal tribes as on *T. dubia* (Thladiantheae) or *B. dioica* (Bryoniae) the pathogen is again more similar to the tribe of origin than to the intermediate tribes, providing evidence that after a certain phylogenetic distance, the correlation between pathogen morphology and phylogenetic position of the host species breaks down. No correlation between sporangial dimension and phylogenetic distance was found.

In summary, the sporangial size and the sporangial shape are of potential value for species delimitation. Sporangiophore length to height of the first ramification and the ratio of the length of the longer to the length of the shorter ultimate branchlet was only weakly affected by host matrix and was invariable and could be of use as well.

The sporangial dimensions and the order of branching were found to be highly dependent on the host matrix. However, these characters impact the infection potential of the next generation, as more often branching sporangiophores bear more sporangia. In addition, it could be assumed that larger sporangia bear more zoospores and can thus produce more potential offspring. *Pseudoperonospora. cubensis* on *Cu. moschata* had only 98 zoospores per sporangiophore, which, in addition to the sparse sporulation (only 0.2 sporangiophores per cm^2^ leaf area), reflects the low susceptibility of the host. *Pseudoperonospora cubensis* on *C. sativus* occupied an intermediate position in almost all characters, but regarding sporangial dimensions and branch orders is in the upper third of the observed values. This leads to a possible number of released zoospores per sporangiophore of 224, which is below the maximum of 350 calculated for *C. anguria*, but with 263.3 sporangiophores per cm^2^ leaf area markedly beyond the pathogen performance on the other hosts. The calculated result of 108.2 sporangia per mm^2^ leaf area is in line with the results of Cohen & Eyal [27], who found a maximum of 102.9 sporangia per mm^2^ (sporulation in darkness, leaf temperature 19.4°C) on *C. sativus* in their investigations. Considering this, *P. cubensis* has a possible mean offspring of 590.1 zoospores per mm^2^ leaf area on *C. sativus*, whereas on *C. anguria* with 49.7 sporangiophores per cm^2^ leaf area it has a mean possible offspring of only 173.8 zoospores per mm^2^ leaf area. Although the highest sporulation density was on *S. angulatus* with 57.3 sporangiophores per mm^2^ sporulating leaf area (*C. sativus* 21.4, *C. anguria* 8.8), the maximum number of zoospores per mm^2^ leaf area is 216.8, due to the relatively low proportion of infected leaf area. In contrast, on *Ci. lanatus P. cubensis* has a mean potential offspring of 3.3 zoospores per mm^2^ and on *Lu. cylindrica* a potential offspring of 16.6 zoospores per mm^2^. The only host on which *P. cubensis* has a higher offspring than on *C. sativus* is *C. melo* with 686.6 zoospores per mm^2^, resulting from sporulation similar in density, but higher in the amount of sporulating leaf area. These findings provide further evidence for the adaptation of the *P. cubensis* strain used in this study to its original host *C. sativus* and the closely related members of *Cucumis*. The estimation of the total amount of infective units per leaf area (Table 3, Figure 3) highlights that there is a huge difference in spore production on the different hosts. It is conceivable that under natural conditions, the infection potential on some hosts is so low that infections would very seldom be found in nature, which might explain the lack of reports of natural infections of *B. dioica* [35], as well as *Cy. pedata* and *T. dubia*.

Oospores could not be investigated in this study, because oospores in *P. cubensis* are usually rare. The only reported occurrence of oospores from *P. cubensis* in Central Europe is in leaves of greenhouse cucumbers in Austria [58]. Despite a field observation of oospores in leaves of cucumber in southern Germany (Runge, unpublished data), the occurrence of oospores seem to be very rare, and the strain used does not readily produce oospores under laboratory conditions and is thus possibly heterothallic. In contrast Cohen et al. [59] have recently reported the massive formation of oospores using two distinct races of *P. cubensis* from Israel. However, when present, oospores could be a useful character in description of species, which have so far not been used frequently for the delimitation of downy mildew species. However, in the genus *Albugo* oospores have been found to be the most important characteristic for species delimitation [15], [16], [19], [60], [61] and it could thus be promising to evaluate oospore characteristics for species delimitation in future studies on Peronosporaceae with wider host spectra. Candidates for this research are *Pseudoperonospora humuli*, which is also able to infect a variety of hosts under laboratory conditions [62], as well as *P. celtidis* [63] and *P. urticae*, as oospore production is abundant in these related pathogens. Taking into consideration the findings of Cohen et al. [59] that, provided the right compatible strains or mating types of *P. cubensis* are used, oospores could be formed abundantly, it could be taken into consideration to do further investigations on the relationship of the sister-species *P. humuli* and *P. cubensis* and the other phylogenetic lineages of the *P. cubensis* species cluster [21], based on oospore morphology.
